# Association between the Chinese diet balance index 2022 and the risk of metabolic syndrome: a cross-sectional analysis in middle-aged and older adults

**DOI:** 10.3389/fnut.2026.1858120

**Published:** 2026-08-07

**Authors:** Yitong Li, Xiaoting Lu, Yu Zeng, Hongshi Wu, Shaojun Huang, Yiqin Qi, Na Li, Fengyi He, Xiuhong Lin, Diaozhu Lin, Ping Liang, Li Yan, Cheng Wang, Min Xia, Chaogang Chen

**Affiliations:** 1 School of Public Health, Sun Yat-sen University, Guangzhou, China; 2 Department of Clinical Nutrition, Sun Yat-sen Memorial Hospital, Sun Yat-sen University, Guangzhou, China; 3 Department of Endocrinology, Sun Yat-sen Memorial Hospital, Sun Yat-sen University, Guangzhou, China

**Keywords:** Chinese diet balance index 2022, diet quality, dietary imbalance, dietary inadequacy, metabolic syndrome

## Abstract

Metabolic syndrome (MetS) represents a major public health challenge, yet evidence regarding the relationship between diet quality, as evaluated by the Chinese diet balance index 2022 (DBI-22), and the risk of MetS remains limited. This cross-sectional study aimed to investigate the association between DBI-22 and MetS among middle-aged and older adults. 3,503 participants aged ≥ 45 years in Guangzhou, China, were included. Dietary intake was assessed using 3-day 24-h records, from which the DBI-22 indicators, including high bound score (HBS), low bound score (LBS), and diet quality distance (DQD), were categorized into two tiers. MetS was defined according to the Joint Interim Statement. Multivariable logistic regression was employed to estimate odds ratios (ORs) and 95% confidence intervals (CIs), and restricted cubic splines were used to examine nonlinear associations. Compared to suitable LBS, unsuitable LBS was associated with higher odds of MetS (OR = 1.28, 95% CI: 1.07–1.54; *p*-trend = 0.008). Unsuitable DQD was associated with higher odds of MetS (OR = 1.28, 95% CI: 1.07–1.52; *p*-trend = 0.006), elevated waist circumference (OR = 1.24, 95% CI: 1.02–1.50; *p*-trend = 0.031), and elevated blood pressure (OR = 1.20, 95% CI: 1.03–1.40; *p*-trend = 0.020). A nonlinear relationship was identified between LBS and low HDL-C (*p*-nonlinear = 0.034), with an inflection point of 17.8. Participants with MetS showed higher rates of severe dietary inadequacy and imbalance. These findings suggested that dietary insufficiency, rather than excess, was significantly associated with MetS, emphasizing the importance of promoting protective food intake in this population.

## 1 Introduction

Metabolic syndrome (MetS) is a cluster of cardiometabolic abnormalities characterized by obesity, hyperglycaemia, dyslipidaemia (elevated triglyceride levels and/or reduced high-density lipoprotein cholesterol levels) and hypertension, posing a substantial threat to public health (1). Crucially, MetS significantly elevates the risk of all-cause and cardiovascular mortality (2). Epidemiological data indicate a global prevalence of 20–25%, underscoring an escalating concern for population health outcomes (3). Among modifiable risk factors, diet is widely recognized as a key determinant in the development and progression of MetS (4).

To quantify adherence to evidence-based dietary recommendations, several *a priori* dietary indices have been developed as invaluable tools for assessing overall diet quality. Commonly used indices include the Mediterranean Diet Score (MDS), Healthy Eating Index (HEI), and the Dietary Approaches to Stop Hypertension (DASH) diet score, all of which have been widely applied to evaluate diet quality and their associations with MetS (5–7). Previous studies from Western populations consistently indicated that these indices correlated with a lower prevalence of MetS (8–10). However, the direct applicability of these indexes to Chinese populations was questionable. China is currently experiencing a nutrition transition characterized by the dual burden of undernutrition and overnutrition. This is reflected in insufficient intakes of dairy, fruits, and soy products, coupled with excessive consumption of refined grains, oils, and salt (11). Therefore, a culturally-specific and validated dietary assessment tool is essential to accurately assess diet quality and its health implications within the Chinese context.

The Chinese diet balance index 2022 (DBI-22) is a recently updated and officially recommended instrument, developed in accordance with the Chinese Dietary Guidelines 2022 and the Chinese Food Guide Pagoda 2022 (12). Previous studies employing earlier versions of the DBI have demonstrated significant associations with various health conditions, including type 2 diabetes, glioma, and sarcopenia (13–15). Distinct from other indices, the DBI-22 was specifically designed to reflect dietary characteristics of Chinese populations and has been validated as a pertinent tool for investigating diet-health relationships in China (16). While previous studies have explored dietary patterns and MetS in China (17), research utilizing the comprehensive and culturally tailored DBI-22 remains limited.

Accordingly, in this community-based, cross-sectional study among middle-aged and older adults, dietary quality was assessed using the DBI-22. The associations of DBI-22 scores with prevalence of MetS and its individual components were investigated.

## 2 Materials and methods

### 2.1 Study population

**Figure 1:**
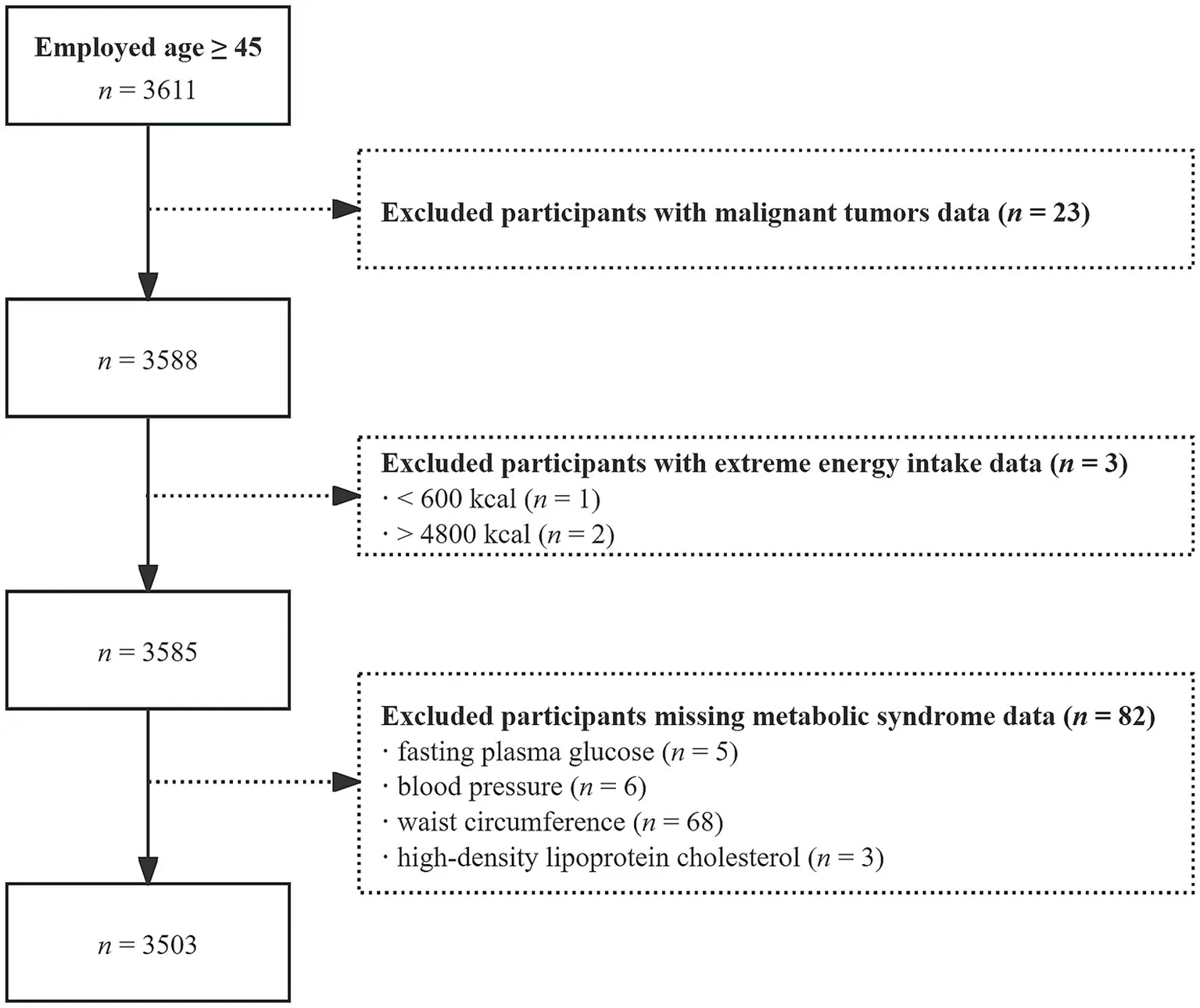
Inclusion/exclusion criteria flow diagram.

This study utilized cross-sectional baseline data from a community-based prospective cohort study in Guangzhou, China, which was designed to investigate the risks of cancer and diabetes among Chinese adults (18). The study design has been described in detail previously (19). In brief, eligible participants aged ≥ 45 years were recruited who had been living in Guangzhou for at least three years. Following the exclusion of individuals with malignant tumors, extreme energy intake (<600 or >4,800 kcal/day), and missing measurement data of fasting plasma glucose (FPG), blood pressure (BP), waist circumference (WC) and high-density lipoprotein cholesterol (HDL-C). A final analytical sample of 3,503 participants aged ≥ 45 years was obtained (Figure 1).

### 2.2 Dietary data collection

Dietary intake data were collected using a 3-day 24-h records method, including two weekdays and one weekend day. Participants were instructed to record all foods and beverages consumed during each 24-h period, with encouragement to document intake in real-time to enhance accuracy. Trained dietitians conducted face-to-face interviews to provide detailed instructions and assist participants in accurately reporting food types, preparation methods, and portion sizes. Standardized food models, household measuring utensils, and visual aids were used to improve the estimation of portion sizes. All dietary records were reviewed for completeness and accuracy by nutrition professionals, with interviewers prompting for any potentially omitted items. Finally, all reported foods and beverages items were classified into predefined food groups according to the China Food Composition Tables (20).

### 2.3 Definition of DBI-22

The Chinese diet balance index 2022 recognized as a prominent dietary assessment tool in China, is recommended for evaluating the overall diet quality of Chinese residents. The DBI-22, an updated version based on the Chinese Dietary Guidelines 2022 and the Chinese Food Guide Pagoda 2022, allows for more accurate evaluation of the sufficiency and diversity of personal dietary intake.

The DBI-22 provides dietary recommendations for each of the 11 energy levels. The scoring system integrates eight core components, encompassing 14 major food groups, assigned scores ranging from −12 to 12. These groups are: (1) cereals; (2) vegetables; (3) fruits; (4) dairy products; (5) soybeans and soy products; (6) red meats, processed meats, poultry, and game; (7) fish and shellfish; (8) eggs; (9) cooking oils; (10) alcoholic beverages; (11) added sugars; (12) salt; (13) dietary variety; (14) water intake. A score of zero indicates intake meeting the recommendation, while positive and negative scores reflect dietary overconsumption and underconsumption, respectively.

Three primary indicators were derived from the component scores: low bound score (LBS), high bound score (HBS) and diet quality distance (DQD). The HBS was calculated as the summation of all positive component scores, quantifying the extent of dietary excess. The LBS was calculated as the absolute value of the sum of all negative component scores, reflecting the magnitude of dietary inadequacy. The DQD was calculated as the absolute values of all individual component scores, providing a composite measure of overall dietary imbalance severity. Due to unavailable data of water intake, the theoretical ranges in this study for HBS, LBS, and DQD were 0–44, 0–60, and 0–84, respectively.

Each indicator was categorized into two tiers based on severity: suitable (mild problem, scores below 40%) and unsuitable (severe problem, scores above 40%). Specific cutoff values were HBS: suitable (0–18), unsuitable (≥19); LBS: suitable (0–24), unsuitable (≥25); DQD: suitable (0–34), unsuitable (≥35). Details of these standards have been reported previously (12).

### 2.4 Definition of metabolic syndrome

Metabolic syndrome (MetS) was defined according to the harmonized criteria established by the Joint Interim Statement (21). Diagnosis required the presence of at least three of the following five components: (1) elevated waist circumference (WC ≥ 90 cm for males or ≥ 80 cm for females); (2) elevated blood pressure (systolic BP ≥ 130 mmHg or diastolic BP ≥ 85 mmHg); (3) low high-density lipoprotein cholesterol (HDL-C < 1.0 mmol/L for males or < 1.3 mmol/L for females); (4) elevated triglycerides (TG ≥ 1.7 mmol/L); (5) elevated fasting plasma glucose (FPG ≥ 5.6 mmol/L).

### 2.5 Covariates

Information on sociodemographic characteristics and lifestyle behaviors was collected via a standardized questionnaire. Education levels were categorized as less than high school, high school, and college or above. Occupation was classified as light, medium and heavy physical labor. Participants were defined as smokers or regular alcohol consumers if they had smoked or consumed alcohol regularly within the past six months. Tea drinkers were identified as those consuming tea at least once per week. Anthropometric measurements, including height and weight, were obtained by trained staff using standardized procedures. Body mass index (BMI) was calculated as weight in kilograms divided by height in meters squared (kg/m^2^).

### 2.6 Statistical analysis

Statistical analyses were performed using R software (version 4.3.1). DBI-22 component scores were calculated based on the established scoring protocol. Descriptive statistics were reported as mean ± standard deviation (SD) for continuous variables and as count (percentage) for categorical variables. Differences in baseline characteristics across DBI-22 indicators tiers were assessed using one-way ANOVA for continuous variables and Chi-square tests for categorical variables.

Multivariable logistic regression analyses were employed to examine the associations between DBI-22 indicators and the prevalence of MetS and its components. Three models were constructed: Model 1 (unadjusted), Model 2 (adjusted for sex, age, education, and occupation), and Model 3 (additionally adjusted for smoking, alcohol consumption, tea drinking, and BMI). Results were expressed as odds ratios (ORs) with 95% confidence intervals (CIs), using the first tier of each DBI-22 indicator as reference. Restricted Cubic Spline (RCS) was used to evaluate potential nonlinear dose–response relationships between continuous DBI-22 scores and MetS and its components. Stratified analyses were conducted across subgroups based on sex, age, BMI, education, occupation, and lifestyle factors and multiplicative interactions were evaluated. Multiple imputation with chained equations was applied to handle missing covariate data. Sensitivity analyses were conducted to assess the robustness of the main findings. To evaluate potential over-adjustment related to adiposity measures, regression models were re-estimated with BMI excluded as a covariate (Supplementary Table S1). Furthermore, complete-case analyses were performed by excluding participants with missing covariate data to examine the potential influence of missing data handling (Supplementary Table S2). A two-sided *p* < 0.05 was considered statistically significant.

## 3 Results

### 3.1 Descriptions of study population

The 3,503 participants had a mean age of 57.43 ± 7.22 years, and 73.6% were female (Table 1). Participants with an unsuitable LBS included a higher proportion of males, had lower education levels, higher rates of smoking and tea consumption, as well as elevated BMI, TG, WC, BP, and reduced HDL-C (all *p* < 0.05). No significant differences were observed for age, occupation, alcohol drinking status and FPG across LBS tiers (all *p* > 0.05). Participants with an unsuitable HBS included lower education levels and higher smoking rates (all *p* < 0.05). Other characteristics did not differ significantly across HBS tiers (all *p* > 0.05). Similarly, an unsuitable DQD were characterized by more males, lower education levels, heavier labor occupation, higher smoking and tea consumption, elevated BMI, TG, WC, BP, and reduced HDL-C (all *p* < 0.05). Age, alcohol consumption status, and FPG did not significantly differ among DQD (all *p* > 0.05).

**Table 1:** The characteristics of the participants stratified by DBI-22^1^.

	All	LBS^2^		*p*	HBS^3^		*p*	DQD^4^		*p*
		Suitable	Unsuitable		Suitable	Unsuitable		Suitable	Unsuitable	
*n*	3,503	2,657	846		1834	1,669		1,174	2,329	
Age, years	57.43 ± 7.22	57.32 ± 6.96	57.78 ± 7.99	0.113	57.48 ± 7.09	57.38 ± 7.37	0.681	57.13 ± 6.34	57.59 ± 7.62	0.077
Sex, *n* (%)				<0.001			0.861			<0.001
Male	925 (26.4)	625 (23.5)	300 (35.5)		482 (26.3)	443 (26.5)		242 (20.6)	683 (29.3)	
Female	2,578 (73.6)	2,032 (76.5)	546 (64.5)		1,352 (73.7)	1,226 (73.5)		932 (79.4)	1,646 (70.7)	
Education levels, *n* (%)				0.005			0.001			<0.001
Less than high school	1,357 (38.7)	989 (37.2)	368 (43.5)		658 (35.9)	699 (41.9)		375 (31.9)	982 (42.2)	
High school	1,862 (53.2)	1,449 (54.5)	413 (48.8)		1,019 (55.6)	843 (50.5)		688 (58.6)	1,174 (50.4)	
College or above	284 (8.1)	219 (8.2)	65 (7.7)		157 (8.6)	127 (7.6)		111 (9.5)	173 (7.4)	
Occupation, *n* (%)				0.181			0.272			0.032
Light labor	2,785 (79.5)	2,130 (80.2)	655 (77.4)		1,476 (80.5)	1,309 (78.4)		963 (82.0)	1,822 (78.2)	
Medium labor	383 (10.9)	285 (10.7)	98 (11.6)		187 (10.2)	196 (11.7)		112 (9.5)	271 (11.6)	
Heavy labor	335 (9.6)	242 (9.1)	93 (11.0)		171 (9.3)	164 (9.8)		99 (8.4)	236 (10.1)	
Smoking status, *n* (%)				<0.001			0.023			<0.001
Non-smoker	2,940 (83.9)	2,288 (86.1)	652 (77.1)		1,564 (85.3)	1,376 (82.4)		1,046 (89.1)	1,894 (81.3)	
Smoker	563 (16.1)	369 (13.9)	194 (22.9)		270 (14.7)	293 (17.6)		128 (10.9)	435 (18.7)	
Alcohol drinking status, *n* (%)				0.083			0.855			0.151
Non-alcohol drinker	2,585 (73.8)	1,980 (74.5)	605 (71.5)		1,351 (73.7)	1,234 (73.9)		884 (75.3)	1,701 (73.0)	
Alcohol drinker	918 (26.2)	677 (25.5)	241 (28.5)		483 (26.3)	435 (26.1)		290 (24.7)	628 (27.0)	
Tea drinking status, *n* (%)				0.011			0.309			0.007
Non-drinker	1,782 (50.9)	1,384 (52.1)	398 (47.0)		948 (51.7)	834 (50.0)		635 (54.1)	1,147 (49.2)	
Drinker	1,721 (49.1)	1,273 (47.9)	448 (53.0)		886 (48.3)	835 (50.0)		539 (45.9)	1,182 (50.8)	
BMI, kg/m^2^	23.20 ± 3.12	23.07 ± 3.07	23.63 ± 3.26	<0.001	23.21 ± 3.15	23.19 ± 3.10	0.850	22.98 ± 3.04	23.32 ± 3.16	0.002
TG, mmol/L	1.55 ± 1.16	1.52 ± 1.14	1.65 ± 1.21	0.003	1.53 ± 1.11	1.58 ± 1.21	0.193	1.46 ± 1.01	1.59 ± 1.23	0.002
FPG, mmol/L	5.72 ± 1.37	5.70 ± 1.34	5.77 ± 1.45	0.186	5.74 ± 1.42	5.70 ± 1.31	0.439	5.69 ± 1.35	5.73 ± 1.37	0.409
HDL-C, mmol/L	1.31 ± 0.37	1.32 ± 0.37	1.28 ± 0.35	0.001	1.31 ± 0.38	1.31 ± 0.35	0.986	1.34 ± 0.39	1.30 ± 0.36	<0.001
WC, cm	80.63 ± 9.15	80.09 ± 9.01	82.33 ± 9.36	<0.001	80.46 ± 9.09	80.83 ± 9.20	0.233	79.50 ± 8.96	81.20 ± 9.19	<0.001
SBP, mmHg	126.37 ± 16.39	125.92 ± 16.22	127.78 ± 16.82	0.004	126.40 ± 16.44	126.33 ± 16.33	0.891	124.98 ± 15.97	127.07 ± 16.55	<0.001
DBP, mmHg	75.07 ± 9.68	74.77 ± 9.66	76.00 ± 9.70	0.001	74.97 ± 9.50	75.18 ± 9.88	0.523	74.11 ± 9.36	75.56 ± 9.81	<0.001

^1^Data are mean ± SD or *n* (%).

^2^LBS: Suitable, score of 0 ~ 24; Unsuitable, score of ≥ 25.

^3^HBS: Suitable, score of 0 ~ 18; Unsuitable, score of ≥ 19.

^4^DQD: suitable, score of 0 ~ 34; unsuitable, score of ≥ 35.

LBS, low bound score; HBS, high bound score; DQD, diet quality distance; BMI, body mass index; TG, triglycerides; FPG, fasting plasma glucose; HDL-C, high-density lipoprotein cholesterol; WC, waist circumference; SBP, systolic blood pressure; DBP, diastolic blood pressure.

### 3.2 Distribution of dietary quality

The intake distribution for DBI-22 components, expressed as the proportions of participants with intakes below, meeting, and above recommendations, is summarized in Table 2. The intake distribution for DBI-22 components was detailed in Supplementary Table S3. Overall, the proportion of participants with MetS who met the recommended intake level varied widely across food groups, ranging from 0.5% (salt) to 99.6% (alcohol). In the MetS group, below recommendation was most notable for dairy products (98.9%), soybeans (78.6%), fruits (77.0%), eggs (66.2%), vegetables (47.2%), fish (30.2%), meat (4.7%), and cereals (0.8%). Among MetS participants, none met the recommended level for dietary diversity (0.0% meet), and 100.0% were below the recommendation. Conversely, above recommendation was observed for salt (99.5%), cereals (95.9%), meat (88.8%), cooking oil (35.1%), eggs (20.5%), and alcoholic beverages (0.5%).

**Table 2:** Proportions of participants with intakes below, meeting, and above recommendations for each DBI-22 component.

Components	Group	Below recommendation (%)	Meet recommendation (%)	Above recommendation (%)
Cereals	Non-MetS	0.9	3.6	95.4
	MetS	0.8	3.4	95.9
Vegetables	Non-MetS	48.6	51.5	-^1^
	MetS	47.2	52.8	-
Fruits	Non-MetS	73.2	26.7	-
	MetS	77.0	23.1	-
Dairy	Non-MetS	98.2	1.8	-
	MetS	98.9	1.2	-
Soybean	Non-MetS	75.4	24.6	-
	MetS	78.6	21.4	-
Meat	Non-MetS	3.7	6.1	90.2
	MetS	4.7	6.6	88.8
Fish	Non-MetS	30.5	69.6	0.0
	MetS	30.2	69.8	0.0
Egg	Non-MetS	63.9	15.7	20.3
	MetS	66.2	13.3	20.5
Oil	Non-MetS	-	68.6	31.4
	MetS	-	64.9	35.1
Salt	Non-MetS	-	0.7	99.4
	MetS	-	0.5	99.5
Alcohol	Non-MetS	-	99.4	0.6
	MetS	-	99.6	0.5
Diet variety	Non-MetS	99.7	0.2	-
	MetS	100.0	0.0	-

^1^″—”: Corresponding evaluation dimension does not apply, as the component score range does not include values in that direction.

MetS, metabolic syndrome.

As shown in Table 3, significant differences were observed in the distribution of LBS and DQD between participants with and without MetS (all *p* < 0.001). A higher proportion of individuals with MetS were classified into the tier representing severe dietary inadequacy (28.7% vs. 22.0%) and severe overall dietary imbalance (71.3% vs. 64.2%), compared to those without MetS. In contrast, the distribution of severe dietary excess tiers did not differ significantly between the two groups (*p* = 0.168).

**Table 3:** Distribution of diet quality and the percentages of participants with each category.

Diet quality	Indicator	Score range	Group	Mean ± SD	Distribution of diet quality (%)		*p*
					Suitable	Unsuitable	
Under intake	LBS^1^	0 ~ 60	Non-MetS	19.59 ± 6.32	78.0	22.0	<0.001
			MetS	20.89 ± 6.21	71.3	28.7	
Over intake	HBS^2^	0 ~ 44	Non-MetS	17.33 ± 4.10	53.2	46.8	0.168
			MetS	17.61 ± 4.11	50.7	49.3	
Overall unbalance	DQD^3^	0 ~ 84	Non-MetS	36.92 ± 8.00	35.8	64.2	<0.001
			MetS	38.50 ± 8.00	28.7	71.3	

^1^LBS: Suitable, score of 0 ~ 24; Unsuitable, score of ≥ 25.

^2^HBS: Suitable, score of 0 ~ 18; Unsuitable, score of ≥ 19.

^3^DQD: suitable, score of 0 ~ 34; unsuitable, score of ≥ 35.

LBS, low bound score; HBS, high bound score; DQD, diet quality distance; MetS, metabolic syndrome.

### 3.3 Associations of DBI-22 with the risk of metabolic syndrome and components

Odds ratios (ORs) and 95% confidence intervals (CIs) for MetS and its components across tiers of LBS, HBS, and DQD were presented in Table 4. After adjusting for age, sex, education, occupation, smoking, alcohol consumption, tea drinking, and BMI, participants with an unsuitable LBS showed a significantly higher odds of MetS compared to those with a suitable LBS (OR = 1.28, 95% CI: 1.07–1.54; *p*-trend = 0.008). However, no significant associations were observed between LBS and individual components of MetS (all *p*-trend > 0.05). Similarly, an unsuitable DQD was associated with higher odds of MetS (OR = 1.28, 95% CI: 1.07–1.52; *p*-trend = 0.006), elevated WC (OR = 1.24, 95% CI: 1.02–1.50; *p*-trend = 0.031), and elevated BP (OR = 1.20, 95% CI: 1.03–1.40; *p*-trend = 0.020). No significant associations were found between DQD and other MetS components (all *p*-trend > 0.05). In contrast, HBS was not significantly associated with MetS or any of its components across all adjusted models (all *p*-trend > 0.05).

**Table 4:** The odds ratios and 95% CIs of metabolic syndrome and components, based on the levels of the DBI-22.

	LBS^1^		*p*-trend	HBS^2^		*p*-trend	DQD^3^		*p*-trend
	Suitable	Unsuitable		Suitable	Unsuitable		Suitable	Unsuitable	
Metabolic syndrome									
Case/total, *n*	803/2657	324/846		571/1834	556/1669		323/1174	804/2329	
Model 1^4^	1.00 (ref.)	1.43 (1.22, 1.68)	<0.001	1.00 (ref.)	1.10 (0.96, 1.27)	0.168	1.00 (ref.)	1.39 (1.19, 1.62)	<0.001
Model 2^5^	1.00 (ref.)	1.45 (1.23, 1.72)	<0.001	1.00 (ref.)	1.08 (0.94, 1.25)	0.288	1.00 (ref.)	1.36 (1.16, 1.59)	<0.001
Model 3^6^	1.00 (ref.)	1.28 (1.07, 1.54)	0.008	1.00 (ref.)	1.11 (0.95, 1.30)	0.203	1.00 (ref.)	1.28 (1.07, 1.52)	0.006
Elevated WC									
Case/total, *n*	1084/2657	397/846		776/1834	705/1669		455/1174	1026/2329	
Model 1^4^	1.00 (ref.)	1.28 (1.10, 1.50)	0.002	1.00 (ref.)	1.00 (0.87, 1.14)	0.966	1.00 (ref.)	1.24 (1.08, 1.44)	0.003
Model 2^5^	1.00 (ref.)	1.44 (1.22, 1.69)	<0.001	1.00 (ref.)	0.97 (0.85, 1.12)	0.704	1.00 (ref.)	1.31 (1.13, 1.52)	<0.001
Model 3^6^	1.00 (ref.)	1.19 (0.96, 1.48)	0.105	1.00 (ref.)	0.98 (0.82, 1.18)	0.850	1.00 (ref.)	1.24 (1.02, 1.50)	0.031
Elevated BP									
Case/total, *n*	990/2657	368/846		704/1834	654/1669		406/1174	952/2329	
Model 1^4^	1.00 (ref.)	1.30 (1.11, 1.52)	0.001	1.00 (ref.)	1.03 (0.90, 1.18)	0.628	1.00 (ref.)	1.31 (1.13, 1.51)	<0.001
Model 2^5^	1.00 (ref.)	1.23 (1.04, 1.45)	0.013	1.00 (ref.)	1.02 (0.89, 1.17)	0.783	1.00 (ref.)	1.23 (1.05, 1.42)	0.008
Model 3^6^	1.00 (ref.)	1.16 (0.98, 1.37)	0.077	1.00 (ref.)	1.04 (0.90, 1.20)	0.600	1.00 (ref.)	1.20 (1.03, 1.40)	0.020
Elevated FPG									
Case/total, *n*	1073/2657	352/846		741/1834	684/1669		459/1174	966/2329	
Model 1^4^	1.00 (ref.)	1.05 (0.90, 1.23)	0.528	1.00 (ref.)	1.02 (0.89, 1.17)	0.727	1.00 (ref.)	1.10 (0.96, 1.27)	0.176
Model 2^5^	1.00 (ref.)	1.01 (0.86, 1.18)	0.916	1.00 (ref.)	1.01 (0.88, 1.16)	0.879	1.00 (ref.)	1.05 (0.91, 1.22)	0.499
Model 3^6^	1.00 (ref.)	0.94 (0.80, 1.11)	0.470	1.00 (ref.)	1.02 (0.88, 1.17)	0.826	1.00 (ref.)	1.01 (0.87, 1.18)	0.865
Elevated TG									
Case/total, *n*	741/2657	281/846		521/1834	501/1669		306/1174	716/2329	
Model 1^4^	1.00 (ref.)	1.29 (1.09, 1.52)	0.003	1.00 (ref.)	1.08 (0.93, 1.25)	0.295	1.00 (ref.)	1.26 (1.08, 1.47)	0.004
Model 2^5^	1.00 (ref.)	1.24 (1.05, 1.47)	0.013	1.00 (ref.)	1.08 (0.93, 1.25)	0.327	1.00 (ref.)	1.22 (1.04, 1.43)	0.016
Model 3^6^	1.00 (ref.)	1.14 (0.96, 1.36)	0.132	1.00 (ref.)	1.08 (0.93, 1.26)	0.289	1.00 (ref.)	1.16 (0.99, 1.37)	0.068
Low HDL-C									
Case/total, *n*	1022/2657	339/846		720/1834	641/1669		443/1174	918/2329	
Model 1^4^	1.00 (ref.)	1.07 (0.91, 1.25)	0.404	1.00 (ref.)	0.96 (0.84, 1.11)	0.605	1.00 (ref.)	1.07 (0.93, 1.24)	0.335
Model 2^5^	1.00 (ref.)	1.16 (0.98, 1.36)	0.079	1.00 (ref.)	0.95 (0.83, 1.09)	0.500	1.00 (ref.)	1.12 (0.97, 1.30)	0.133
Model 3^6^	1.00 (ref.)	1.07 (0.91, 1.27)	0.396	1.00 (ref.)	0.95 (0.83, 1.10)	0.497	1.00 (ref.)	1.07 (0.92, 1.24)	0.382

^1^LBS: Suitable, score of 0 ~ 24; Unsuitable, score of ≥ 25.

^2^HBS: Suitable, score of 0 ~ 18; Unsuitable, score of ≥ 19.

^3^DQD: suitable, score of 0 ~ 34; unsuitable, score of ≥ 35.

^4^Unadjusted.

^5^Adjusted for age, sex, education levels and occupation.

^6^Adjusted additionally for smoking status, alcohol drinking status, tea drinking status and BMI.

LBS, low bound score; HBS, high bound score; DQD, diet quality distance; WC, waist circumference; BP, blood pressure; FPG, fasting plasma glucose; TG, triglycerides; HDL-C, high-density lipoprotein cholesterol; BMI, body mass index.

### 3.4 Interactions and stratified analyses

**Figure 2:**
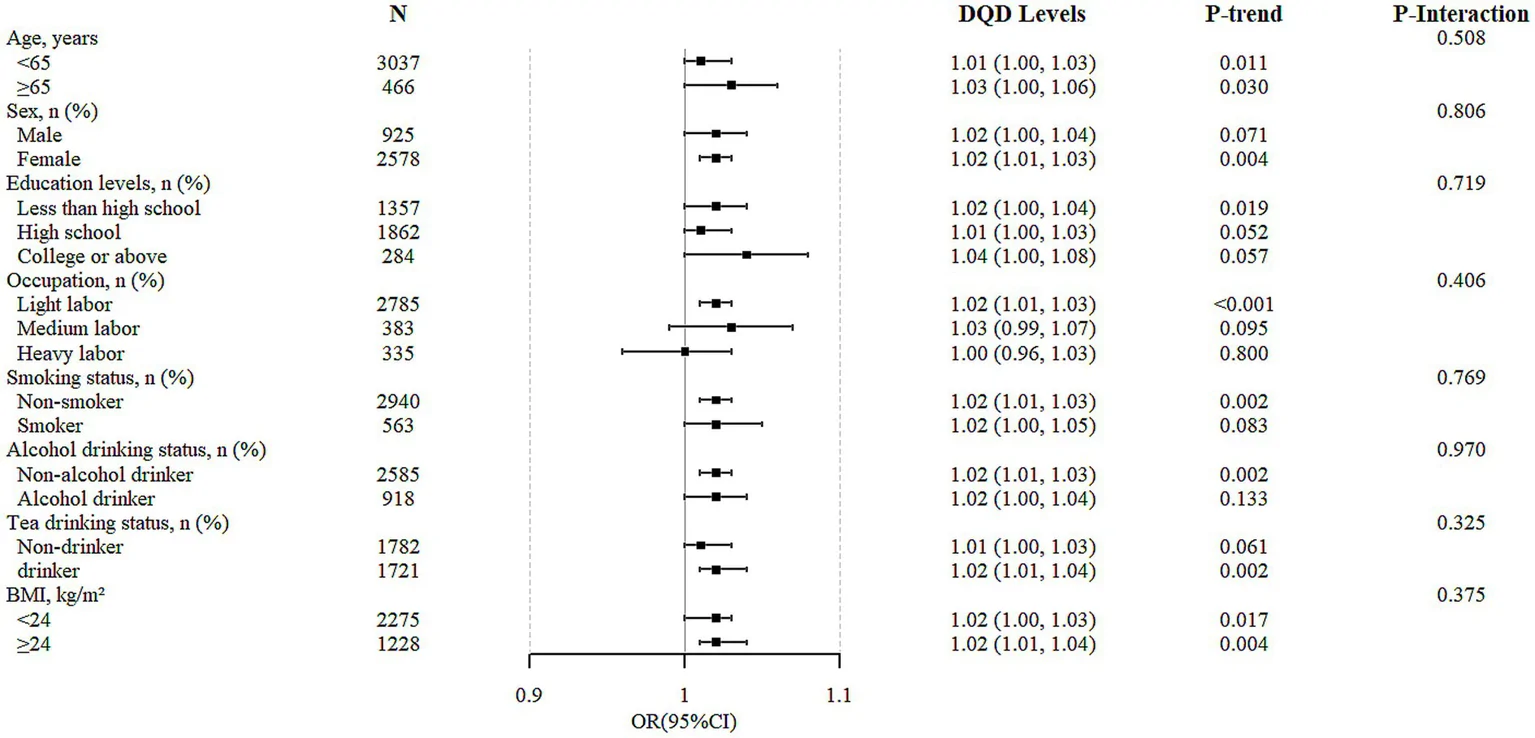
The risk of metabolic syndrome associated with DQD in the subgroup analysis^1^.1 Adjusted for age, sex, education levels, occupation, smoking status, alcohol drinking status, tea drinking status, and BMI. LBS, low bound score; HBS, high bound score; DQD, diet quality distance; BMI, body mass index.

Stratified analyses were conducted to evaluate the association between DQD and the risk of MetS across various subgroups, with adjustment for multiple covariates (Figure 2). No significant multiplicative interactions were identified between DQD and any of the subgroups examined (all *p*-interaction > 0.05). Similar associations were observed for LBS and HBS (Supplementary Figures S1, S2; all *p*-interaction > 0.05).

### 3.5 Nonlinear relationship assessment

**Figure 3:**
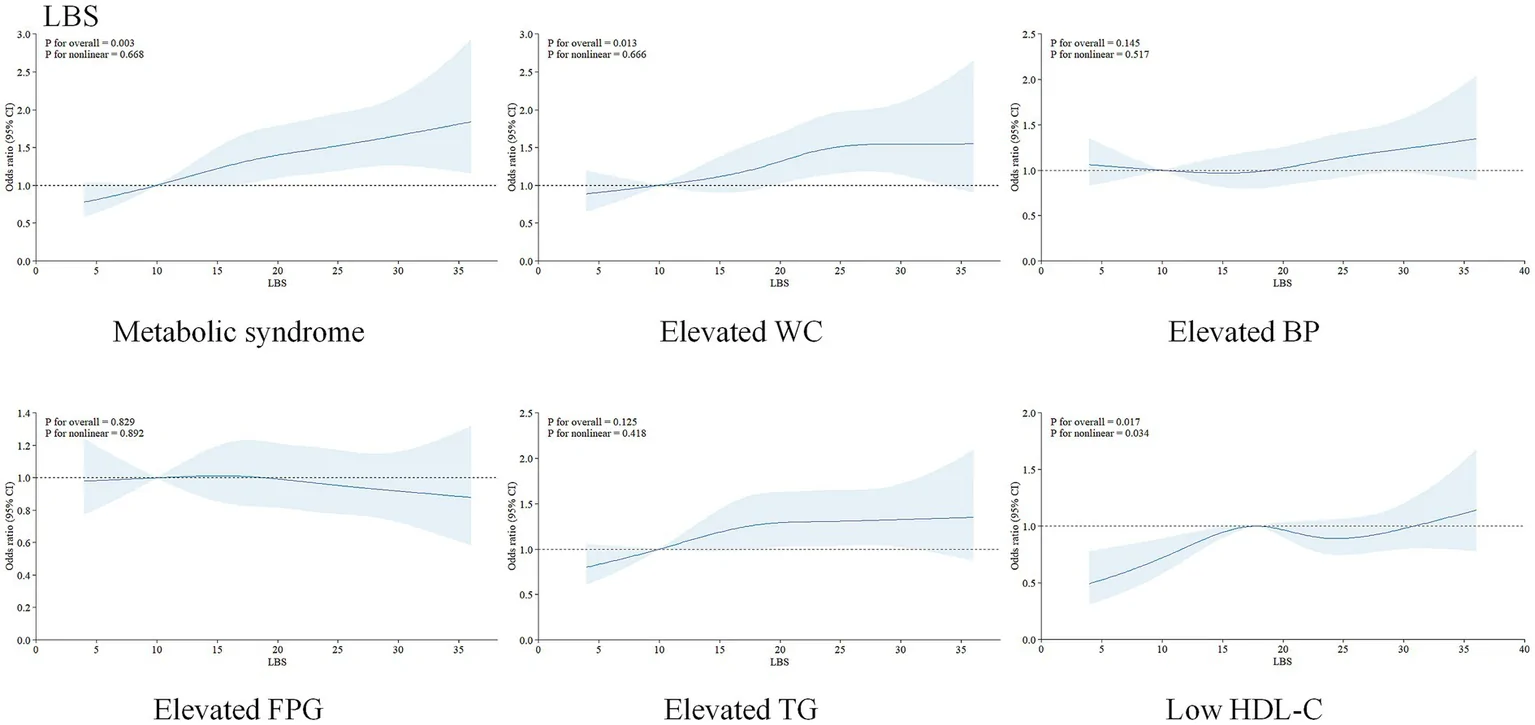
Relation of LBS with the risks of metabolic syndrome. Adjusted for age, sex, education levels, occupation, smoking status, alcohol drinking status, tea drinking status and BMI. LBS, low bound score; WC, waist circumference; BP, blood pressure; FPG, fasting plasma glucose; TG, triglycerides; HDL-C, high-density lipoprotein cholesterol; BMI, body mass index.

Potential nonlinear associations between continuous LBS and MetS and its components were assessed using restricted cubic splines (RCS) (Figure 3). After adjusting for age, sex, education levels, occupation, smoking status, alcohol drinking status, tea drinking status and BMI, a significant L-shaped relationship was identified between LBS and low HDL-C (*p*-overall = 0.017; *p*-nonlinear = 0.034), with an inflection point at 17.8. In contrast, no significant nonlinear associations were observed between LBS and MetS or its other components (all *p*-nonlinear > 0.05). Similarly, fully adjusted RCS models showed no significant nonlinear relationships for either HBS or DQD with MetS or any MetS components (Supplementary Figures S3, S4).

### 3.6 Sensitivity analysis

To assess the robustness and potential confounding of the primary findings, we conducted two sensitivity analyses. First, when BMI was excluded from the fully adjusted models (Models 3) to assess the primary association without potential over-adjustment, the associations between DBI-22 and several metabolic syndrome components were materially altered. Specifically, associations that were not statistically significant in the main analysis became significant after excluding BMI, including elevated WC–LBS (OR = 1.42, 95% CI: 1.21–1.68), elevated BP–LBS (OR = 1.24, 95% CI: 1.05–1.46), elevated TG–LBS (OR = 1.23, 95% CI: 1.04–1.46), and elevated TG–DQD (OR = 1.21, 95% CI: 1.03–1.42) ((Supplementary Table S1)). Second, analyses that incorporated multiple imputation of missing covariates or excluded statistical outliers yielded results that were essentially unchanged from the main models ((Supplementary Table S2)), suggesting that neither missingness nor extreme values materially influenced the findings.

## 4 Discussion

In this cross-sectional study of middle-aged and older adults in Guangzhou, we applied the DBI-22 to evaluate diet quality and its relationship with MetS and its individual components. We found that both unsuitable LBS and DQD were significantly associated with higher odds of MetS. Furthermore, an unsuitable DQD was positively correlated with elevated waist circumference and blood pressure. A nonlinear dose–response relationship was observed between LBS and low HDL-C, with an inflection point at 17.8. Moreover, participants with MetS exhibited moderate to severe dietary imbalance, characterized specifically by insufficient intake of dairy, fruits, and soybeans, alongside excessive consumption of cereals, meat, and salt. The associations between higher LBS and DQD and MetS remained consistent across most subgroups.

The LBS, which reflects insufficient food consumption relative to dietary guidelines, was positively associated with MetS. This pattern was consistent across multiple MetS components, including elevated TG and low HDL-C. The primary contributors to LBS among participants with MetS were inadequate intakes of dairy products, dietary variety, fruit and soybeans. A meta-analysis of 12 studies indicated an inverse relationship between dairy consumption, especially low-fat dairy, milk, and yogurt and the incidence of MetS (22). Similarly, a prospective cohort study in Korea demonstrated that greater dietary diversity was associated with a reduced risk of MetS in men (23). Another cross-sectional study reported significantly lower MetS prevalence among individuals in the highest quartile of fresh fruit consumption compared to those in the lowest quartile (24). Furthermore, research involving Korean women linked moderate-to-high weekly soybean consumption (8.5–17 times per week) with a lower likelihood of MetS (25). These findings collectively support the concept that insufficient intake of dairy products, fruits, and soybeans may be associated with MetS, which aligns with the associations observed in our study. Notably, an L-shaped association was also discovered between LBS and low HDL-C, with a breakpoint of 17.8. Below this threshold, the odds of low HDL-C increased with rising LBS, whereas above the breakpoint, the association plateaued and the upward trend attenuated, suggests that HDL-C may be sensitive to early dietary imbalance. The plateau observed beyond this threshold may indicate a saturation effect, where additional dietary inadequacy does not substantially further worsen HDL-C levels. This finding implies that even mild dietary imbalance may be relevant for lipid metabolism and highlights the importance of early dietary improvement. Importantly, inadequate intake of dairy products and soybeans were the major contributors to elevated LBS. A recent systematic review with network meta-analysis of 19 randomized controlled trials reported that full-fat dairy consumption may increase HDL-C compared with a control diet (26). Similarly, clinical evidence has demonstrated that soybean intake significantly increases HDL-C concentrations in hypercholesterolemic patients (27). These findings indicated that addressing dietary inadequacies, particularly for these key food groups, may help mitigate progressive declines in HDL-C levels.

In contrast to LBS, the HBS showed no significant association with MetS or its individual components in our analysis. This may be attributed to the specific food groups that contributed most substantially to HBS in this study, primarily cereals, meat, and salt. Existing evidence indicated that refined grains were a risk factor for MetS, whereas whole grains were protective (28). Notably, the DBI-22 did not differentiate between whole and refined grains (29). Dietary shift in China has been toward refined grains (30), while whole grain intake remains low (31), which may have diluted the observed association for cereals. Similarly, the DBI-22 combines red meat and poultry into a single category, despite studies showing that red meat intake was positively associated with MetS, whereas poultry intake was inversely associated (32). The aggregation of these heterogenous food groups may have further attenuated the overall effect. For other food groups, such as salt, the relationship with MetS was complex. Although high salt intake was an established risk factor for hypertension, its independent association with other metabolic abnormalities remains inconsistent (33, 34). Therefore, the lack of a significant association between HBS and MetS may be explained by the heterogeneous composition of HBS, as the included food groups have varying and potentially opposite associations with MetS, which may attenuate the overall association in this population.

In the present study, we observed that the inclusion of BMI in the fully adjusted models attenuated several associations between DBI-22 and metabolic syndrome components, particularly waist circumference, blood pressure, triglycerides, and dietary quality–related indicators. When BMI was excluded in sensitivity analyses, several associations that were not statistically significant in the main analysis became significant. This pattern suggests that BMI may capture shared variance with both dietary behavior and metabolic outcomes, potentially leading to conservative effect estimates when included as a covariate. Given that BMI reflects general adiposity and is closely correlated with central obesity and metabolic risk factors, its inclusion in regression models of adiposity-related endpoints may partially obscure underlying associations.

The DQD, which quantifies total deviation from a balanced diet by integrating both insufficient and excessive intake scores, was positively associated with MetS. This finding was consistent with previous studies using diet quality indices based on national or regional dietary guidelines. In a large nationally representative US cohort, better diet quality as measured by the Healthy Eating Index-2015 (HEI-2015) was correlated with decreased odds of MetS (9). Similarly, adherence to the 2017 French dietary guidelines was associated with a lower risk of overweight and obesity, as well as higher HDL-C levels (35, 36). Additionally, research on the Mediterranean diet has demonstrated that greater adherence corresponds to a lower probability of developing MetS (10). These consistent findings across diverse populations and dietary frameworks underscored the importance of adhering to healthy dietary patterns to prevent and mitigate MetS.

Appropriate dietary practices were fundamental to the prevention and management of MetS (37). In light of our findings, we recommend optimizing dietary habits by addressing the specific imbalances identified through the DBI-22 framework. This approach emphasizes not only moderating excessive intakes but also, and more importantly, increasing the consumption of under-consumed protective foods. Following the principles of the Chinese Dietary Guidelines 2022 (38) and the Food Guide Pagoda 2022, individuals should strive to achieve a diverse and nutritionally balanced diet. Adopting these healthy dietary patterns represents a cornerstone strategy for reducing the risk of MetS and promoting long-term metabolic health (39).

This study had several notable strengths. It represents one of the first applications of the updated DBI-22 to evaluate associations with MetS and its components in a well-defined urban Chinese population. A key advantage of the DBI-22 was its ability to separately assess dietary insufficiency and excess through the LBS and HBS, enabling a more nuanced evaluation of diet quality. Furthermore, analyzing MetS both as a composite outcome and through its individual components provided enhanced insight into the relationships between dietary factors and specific metabolic abnormalities.

However, several limitations must be acknowledged. To begin with, the cross-sectional design precludes definitive establishment of causal relationships, and the potential for reverse causation. Furthermore, caution is warranted when generalizing these findings to other populations with different demographic characteristics or dietary customs, as the study was conducted among community-dwelling adults in Guangzhou. Moreover, the sample was disproportionately female, which may introduce bias and limit generalizability despite adjustment for sex in all multivariable models. Additionally, the 3-day 24-h records method was susceptible to random measurement error, which may have led to imprecise capture of episodically consumed items such as added sugars and alcohol, potentially introducing bias. We adjusted for alcohol consumption in our analyses to partially mitigate this concern. Another limitation is that, owing to data unavailability, the DBI-22 scores were calculated excluding the water intake component, which may affect score validity and participant classification. The validated ‘without-water’ version of the DBI-22 was used to partially mitigate this limitation. Additionally, the aggregation of food groups within the DBI-22 may have attenuated the associations between dietary indicators and MetS, as some combined food categories may contain components with heterogeneous metabolic effects. Finally, multiple statistical tests were performed, encompassing analyses of DBI-22 indicators, MetS components, and subgroup and interaction effects. Therefore, the possibility of type I error, especially in subgroup and interaction analyses, cannot be ruled out.

In summary, we observed that higher dietary insufficiency and overall imbalance assessed by DBI-22 were significantly associated with higher odds of MetS in middle-aged and older adults, particularly through elevated WC and BP. In contrast, dietary excess showed no independent association with MetS. Optimizing intake of under-consumed protective foods, including fruits, dairy products and soybeans, may be more relevant for addressing MetS than solely focusing on restricting overconsumption in China population.

## Data Availability

The raw data supporting the conclusions of this article will be made available by the authors, without undue reservation.

## Supplementary material

SUPPLEMENTARY FIGURE S1 The risk of metabolic syndrome associated with LBS in the subgroup analysis. Adjusted for age, sex, education levels, occupation, smoking status, alcohol drinking status, tea drinking status and BMI. LBS, low bound score; BMI, body mass index.

SUPPLEMENTARY FIGURE S2 The risk of metabolic syndrome associated with HBS in the subgroup analysis. Adjusted for age, sex, education levels, occupation, smoking status, alcohol drinking status, tea drinking status and BMI. HBS, high bound score; BMI, body mass index.

SUPPLEMENTARY FIGURE S3 Relation of HBS with the risks of metabolic syndrome. Adjusted for age, sex, education levels, occupation, smoking status, alcohol drinking status, tea drinking status and BMI. HBS, high bound score; WC, waist circumference; BP, blood pressure; FPG, fasting plasma glucose; TG, triglycerides; HDL-C, high-density lipoprotein cholesterol; BMI, body mass index.

SUPPLEMENTARY FIGURE S4 Relation of DQD with the risks of metabolic syndrome. Adjusted for age, sex, education levels, occupation, smoking status, alcohol drinking status, tea drinking status and BMI. DQD, diet quality distance; WC, waist circumference; BP, blood pressure; FPG, fasting plasma glucose; TG, triglycerides; HDL-C, high-density lipoprotein cholesterol; BMI, body mass index.
