# Supplementary material for: Association between the Chinese diet balance index 2022 and the risk of metabolic syndrome: a cross-sectional analysis in middle-aged and older adults
Source: Front Nutr. 2026 Aug 7;13:1858120. doi: 10.3389/fnut.2026.1858120 (PMC13495691; doi:10.3389/fnut.2026.1858120)
Supplement: Supplementary file 1 [file Table_1.docx]

**Table S1** The odds ratios and 95% CIs of metabolic syndrome and components, based on the levels of the DBI-22: sensitivity analysis excluding BMI.

|  | LBS^1^ | | *P*-trend | HBS^2^ | | *P*-trend | DQD^3^ | | *P*-trend |
| --- | --- | --- | --- | --- | --- | --- | --- | --- | --- |
|  | Suitable | Unsuitable |  | Suitable | Unsuitable |  | Suitable | Unsuitable |  |
| ***Metabolic syndrome*** | |  |  |  |  |  |  |  |  |
| Case/total, *n* | 803/2657 | 324/846 |  | 571/1834 | 556/1669 |  | 323/1174 | 804/2329 |  |
| model 1^4^ | 1.00 (ref.) | 1.43 (1.22, 1.68) | <0.001 | 1.00 (ref.) | 1.10 (0.96, 1.27) | 0.168 | 1.00 (ref.) | 1.39 (1.19, 1.62) | <0.001 |
| model 2^5^ | 1.00 (ref.) | 1.45 (1.23, 1.72) | <0.001 | 1.00 (ref.) | 1.08 (0.94, 1.25) | 0.288 | 1.00 (ref.) | 1.36 (1.16, 1.59) | <0.001 |
| model 3^6^ | 1.00 (ref.) | 1.44 (1.22, 1.70) | <0.001 | 1.00 (ref.) | 1.08 (0.93, 1.24) | 0.324 | 1.00 (ref.) | 1.35 (1.15, 1.58) | <0.001 |
| ***Elevated WC*** |  |  |  |  |  |  |  |  |  |
| Case/total, *n* | 1084/2657 | 397/846 |  | 776/1834 | 705/1669 |  | 455/1174 | 1026/2329 |  |
| model 1^4^ | 1.00 (ref.) | 1.28 (1.10, 1.50) | 0.002 | 1.00 (ref.) | 1.00 (0.87, 1.14) | 0.966 | 1.00 (ref.) | 1.24 (1.08, 1.44) | 0.003 |
| model 2^5^ | 1.00 (ref.) | 1.44 (1.22, 1.69) | <0.001 | 1.00 (ref.) | 0.97 (0.85, 1.12) | 0.704 | 1.00 (ref.) | 1.31 (1.13, 1.52) | <0.001 |
| model 3^6^ | 1.00 (ref.) | 1.42 (1.21, 1.68) | <0.001 | 1.00 (ref.) | 0.97 (0.84, 1.11) | 0.643 | 1.00 (ref.) | 1.29 (1.11, 1.50) | 0.001 |
| ***Elevated BP*** |  |  |  |  |  |  |  |  |  |
| Case/total, *n* | 990/2657 | 368/846 |  | 704/1834 | 654/1669 |  | 406/1174 | 952/2329 |  |
| model 1^4^ | 1.00 (ref.) | 1.30 (1.11, 1.52) | 0.001 | 1.00 (ref.) | 1.03 (0.90, 1.18) | 0.628 | 1.00 (ref.) | 1.31 (1.13, 1.51) | <0.001 |
| model 2^5^ | 1.00 (ref.) | 1.23 (1.04, 1.45) | 0.013 | 1.00 (ref.) | 1.02 (0.89, 1.17) | 0.783 | 1.00 (ref.) | 1.23 (1.05, 1.42) | 0.008 |
| model 3^6^ | 1.00 (ref.) | 1.24 (1.05, 1.46) | 0.010 | 1.00 (ref.) | 1.03 (0.89, 1.19) | 0.682 | 1.00 (ref.) | 1.24 (1.07, 1.44) | 0.005 |
| ***Elevated FPG*** | |  |  |  |  |  |  |  |  |
| Case/total, *n* | 1073/2657 | 352/846 |  | 741/1834 | 684/1669 |  | 459/1174 | 966/2329 |  |
| model 1^4^ | 1.00 (ref.) | 1.05 (0.90, 1.23) | 0.528 | 1.00 (ref.) | 1.02 (0.89, 1.17) | 0.727 | 1.00 (ref.) | 1.10 (0.96, 1.27) | 0.176 |
| model 2^5^ | 1.00 (ref.) | 1.01 (0.86, 1.18) | 0.916 | 1.00 (ref.) | 1.01 (0.88, 1.16) | 0.879 | 1.00 (ref.) | 1.05 (0.91, 1.22) | 0.499 |
| model 3^6^ | 1.00 (ref.) | 1.01 (0.86, 1.18) | 0.950 | 1.00 (ref.) | 1.01 (0.88, 1.16) | 0.899 | 1.00 (ref.) | 1.05 (0.91, 1.21) | 0.531 |
| ***Elevated TG*** |  |  |  |  |  |  |  |  |  |
| Case/total, *n* | 741/2657 | 281/846 |  | 521/1834 | 501/1669 |  | 306/1174 | 716/2329 |  |
| model 1^4^ | 1.00 (ref.) | 1.29 (1.09, 1.52) | 0.003 | 1.00 (ref.) | 1.08 (0.93, 1.25) | 0.295 | 1.00 (ref.) | 1.26 (1.08, 1.47) | 0.004 |
| model 2^5^ | 1.00 (ref.) | 1.24 (1.05, 1.47) | 0.013 | 1.00 (ref.) | 1.08 (0.93, 1.25) | 0.327 | 1.00 (ref.) | 1.22 (1.04, 1.43) | 0.016 |
| model 3^6^ | 1.00 (ref.) | 1.23 (1.04, 1.46) | 0.016 | 1.00 (ref.) | 1.07 (0.92, 1.24) | 0.358 | 1.00 (ref.) | 1.21 (1.03, 1.42) | 0.020 |
| ***Low HDL-C*** |  |  |  |  |  |  |  |  |  |
| Case/total, *n* | 1022/2657 | 339/846 |  | 720/1834 | 641/1669 |  | 443/1174 | 918/2329 |  |
| model 1^4^ | 1.00 (ref.) | 1.07 (0.91, 1.25) | 0.404 | 1.00 (ref.) | 0.96 (0.84, 1.11) | 0.605 | 1.00 (ref.) | 1.07 (0.93, 1.24) | 0.335 |
| model 2^5^ | 1.00 (ref.) | 1.16 (0.98, 1.36) | 0.079 | 1.00 (ref.) | 0.95 (0.83, 1.09) | 0.500 | 1.00 (ref.) | 1.12 (0.97, 1.30) | 0.133 |
| model 3^6^ | 1.00 (ref.) | 1.14 (0.97, 1.35) | 0.107 | 1.00 (ref.) | 0.94 (0.82, 1.09) | 0.421 | 1.00 (ref.) | 1.11 (0.95, 1.28) | 0.184 |

^1^ LBS: Suitable, score of 0 ~ 24; Unsuitable, score of ≥ 25. ^2^ HBS: Suitable, score of 0 ~ 18; Unsuitable, score of ≥ 19. ^3^ DQD: suitable, score of 0 ~ 34; unsuitable, score of ≥ 35.

^4^ Unadjusted. ^5^ Adjusted for age, sex, education levels and occupation. ^6^ Adjusted additionally for smoking status, alcohol drinking status and tea drinking status.

Abbreviations: LBS, low bound score; HBS, high bound score; DQD, diet quality distance; WC, waist circumference; BP, blood pressure; FPG, fasting plasma glucose; TG, triglycerides; HDL-C, high-density lipoprotein cholesterol; BMI, body mass index.
