# Supplementary material for: Association between the Chinese diet balance index 2022 and the risk of metabolic syndrome: a cross-sectional analysis in middle-aged and older adults
Source: Front Nutr. 2026 Aug 7;13:1858120. doi: 10.3389/fnut.2026.1858120 (PMC13495691; doi:10.3389/fnut.2026.1858120)
Supplement: Supplementary file 2 [file Table_2.docx]

**Table S2** The odds ratios and 95% CIs of metabolic syndrome and components, based on the levels of the DBI-22: complete-case analysis.

|  | LBS^1^ | | *P*-trend | HBS^2^ | | *P*-trend | DQD^3^ | | *P*-trend |
| --- | --- | --- | --- | --- | --- | --- | --- | --- | --- |
|  | Suitable | Unsuitable |  | Suitable | Unsuitable |  | Suitable | Unsuitable |  |
| ***Metabolic syndrome*** | |  |  |  |  |  |  |  |  |
| Case/total, *n* | 790/2598 | 314/817 |  | 558/1786 | 546/1629 |  | 319/1147 | 785/2268 |  |
| model 1^4^ | 1.00 (ref.) | 1.43 (1.21, 1.68) | <0.001 | 1.00 (ref.) | 1.11 (0.96, 1.28) | 0.156 | 1.00 (ref.) | 1.37 (1.18, 1.61) | <0.001 |
| model 2^5^ | 1.00 (ref.) | 1.46 (1.23, 1.73) | <0.001 | 1.00 (ref.) | 1.08 (0.93, 1.25) | 0.300 | 1.00 (ref.) | 1.34 (1.15, 1.58) | <0.001 |
| model 3^6^ | 1.00 (ref.) | 1.28 (1.06, 1.55) | 0.009 | 1.00 (ref.) | 1.13 (0.96, 1.33) | 0.148 | 1.00 (ref.) | 1.26 (1.06, 1.51) | 0.010 |
| ***Elevated WC*** |  |  |  |  |  |  |  |  |  |
| Case/total, *n* | 1066/2598 | 386/817 |  | 763/1786 | 689/1629 |  | 449/1147 | 1003/2268 |  |
| model 1^4^ | 1.00 (ref.) | 1.29 (1.10, 1.51) | 0.002 | 1.00 (ref.) | 0.98 (0.86, 1.13) | 0.802 | 1.00 (ref.) | 1.23 (1.07, 1.42) | 0.005 |
| model 2^5^ | 1.00 (ref.) | 1.46 (1.24, 1.73) | <0.001 | 1.00 (ref.) | 0.95 (0.83, 1.10) | 0.511 | 1.00 (ref.) | 1.30 (1.12, 1.51) | <0.001 |
| model 3^6^ | 1.00 (ref.) | 1.22 (0.98, 1.53) | 0.074 | 1.00 (ref.) | 0.98 (0.82, 1.18) | 0.847 | 1.00 (ref.) | 1.23 (1.01, 1.49) | 0.041 |
| ***Elevated BP*** |  |  |  |  |  |  |  |  |  |
| Case/total, *n* | 970/2598 | 355/817 |  | 685/1786 | 640/1629 |  | 396/1147 | 929/2268 |  |
| model 1^4^ | 1.00 (ref.) | 1.29 (1.10, 1.51) | 0.002 | 1.00 (ref.) | 1.04 (0.91, 1.19) | 0.576 | 1.00 (ref.) | 1.32 (1.14, 1.53) | <0.001 |
| model 2^5^ | 1.00 (ref.) | 1.22 (1.04, 1.44) | 0.017 | 1.00 (ref.) | 1.02 (0.89, 1.18) | 0.772 | 1.00 (ref.) | 1.23 (1.06, 1.44) | 0.007 |
| model 3^6^ | 1.00 (ref.) | 1.15 (0.97, 1.37) | 0.105 | 1.00 (ref.) | 1.05 (0.91, 1.21) | 0.532 | 1.00 (ref.) | 1.21 (1.03, 1.41) | 0.019 |
| ***Elevated FPG*** | |  |  |  |  |  |  |  |  |
| Case/total, *n* | 1041/2598 | 337/817 |  | 714/1786 | 664/1629 |  | 445/1147 | 933/2268 |  |
| model 1^4^ | 1.00 (ref.) | 1.05 (0.89, 1.23) | 0.549 | 1.00 (ref.) | 1.03 (0.90, 1.18) | 0.641 | 1.00 (ref.) | 1.10 (0.95, 1.27) | 0.188 |
| model 2^5^ | 1.00 (ref.) | 1.01 (0.86, 1.19) | 0.914 | 1.00 (ref.) | 1.02 (0.89, 1.17) | 0.812 | 1.00 (ref.) | 1.05 (0.91, 1.22) | 0.510 |
| model 3^6^ | 1.00 (ref.) | 0.94 (0.79, 1.11) | 0.436 | 1.00 (ref.) | 1.03 (0.89, 1.18) | 0.704 | 1.00 (ref.) | 1.01 (0.87, 1.17) | 0.898 |
| ***Elevated TG*** |  |  |  |  |  |  |  |  |  |
| Case/total, *n* | 725/2598 | 272/817 |  | 509/1786 | 488/1629 |  | 300/1147 | 697/2268 |  |
| model 1^4^ | 1.00 (ref.) | 1.29 (1.09, 1.53) | 0.003 | 1.00 (ref.) | 1.07 (0.93, 1.24) | 0.349 | 1.00 (ref.) | 1.25 (1.07, 1.47) | 0.006 |
| model 2^5^ | 1.00 (ref.) | 1.24 (1.05, 1.47) | 0.013 | 1.00 (ref.) | 1.07 (0.92, 1.24) | 0.395 | 1.00 (ref.) | 1.21 (1.03, 1.42) | 0.020 |
| model 3^6^ | 1.00 (ref.) | 1.14 (0.96, 1.36) | 0.139 | 1.00 (ref.) | 1.08 (0.93, 1.26) | 0.300 | 1.00 (ref.) | 1.16 (0.98, 1.37) | 0.080 |
| ***Low HDL-C*** |  |  |  |  |  |  |  |  |  |
| Case/total, *n* | 1002/2598 | 325/817 |  | 703/1786 | 624/1629 |  | 436/1147 | 891/2268 |  |
| model 1^4^ | 1.00 (ref.) | 1.05 (0.90, 1.24) | 0.535 | 1.00 (ref.) | 0.96 (0.83, 1.10) | 0.527 | 1.00 (ref.) | 1.06 (0.91, 1.22) | 0.471 |
| model 2^5^ | 1.00 (ref.) | 1.14 (0.97, 1.34) | 0.123 | 1.00 (ref.) | 0.94 (0.82, 1.08) | 0.395 | 1.00 (ref.) | 1.09 (0.94, 1.27) | 0.234 |
| model 3^6^ | 1.00 (ref.) | 1.05 (0.89, 1.25) | 0.561 | 1.00 (ref.) | 0.94 (0.82, 1.09) | 0.437 | 1.00 (ref.) | 1.04 (0.90, 1.22) | 0.585 |

^1^ LBS: Suitable, score of 0 ~ 24; Unsuitable, score of ≥ 25. ^2^ HBS: Suitable, score of 0 ~ 18; Unsuitable, score of ≥ 19. ^3^ DQD: suitable, score of 0 ~ 34; unsuitable, score of ≥ 35.

^4^ Unadjusted. ^5^ Adjusted for age, sex, education levels and occupation. ^6^ Adjusted additionally for smoking status, alcohol drinking status, tea drinking status and BMI.

Abbreviations: LBS, low bound score; HBS, high bound score; DQD, diet quality distance; WC, waist circumference; BP, blood pressure; FPG, fasting plasma glucose; TG, triglycerides; HDL-C, high-density lipoprotein cholesterol; BMI, body mass index.
