# Supplementary material for: Association between the Chinese diet balance index 2022 and the risk of metabolic syndrome: a cross-sectional analysis in middle-aged and older adults
Source: Front Nutr. 2026 Aug 7;13:1858120. doi: 10.3389/fnut.2026.1858120 (PMC13495691; doi:10.3389/fnut.2026.1858120)
Supplement: Supplementary file 3 [file Table_3.docx]

**Table S3** Distribution of scores for the DBI-22 components and the percentage of participants with each score.

| Components | Score range | Group | Distribution of scores (%) | | | | | | | | | | | | | *P* |
| --- | --- | --- | --- | --- | --- | --- | --- | --- | --- | --- | --- | --- | --- | --- | --- | --- |
|  |  |  | (-12)- (-11) | (-10)- (-9) | (-8)- (-7) | (-6)- (-5) | (-4)- (-3) | (-2)- (-1) | 0 | (1)- (2) | (3)- (4) | (5)- (6) | (7)- (8) | (9)- (10) | (11)- (12) |  |
| Cereals | (−12) - (12) | Non-MetS | 0.0 | 0.1 | 0.0 | 0.1 | 0.2 | 0.5 | 3.6 | 2.9 | 2.8 | 4.5 | 4.8 | 3.8 | 76.6 | 0.946 |
|  |  | MetS | 0.0 | 0.0 | 0.2 | 0.0 | 0.2 | 0.4 | 3.4 | 2.6 | 3.1 | 4.8 | 4.2 | 3.8 | 77.4 |  |
| Vegetables | (−6) - (0) | Non-MetS | - | - | - | 0.5 | 13.3 | 34.8 | 51.5 | - | - | - | - | - | - | 0.317 |
|  |  | MetS | - | - | - | 0.8 | 14.1 | 32.3 | 52.8 | - | - | - | - | - | - |  |
| Fruits | (−6) - (0) | Non-MetS | - | - | - | 26.4 | 25.8 | 21.0 | 26.7 | - | - | - | - | - | - | 0.007 |
|  |  | MetS | - | - | - | 31.5 | 24.0 | 21.5 | 23.1 | - | - | - | - | - | - |  |
| Dairy | (−6) - (0) | Non-MetS | - | - | - | 51.5 | 35.5 | 11.2 | 1.8 | - | - | - | - | - | - | <0.001 |
|  |  | MetS | - | - | - | 60.9 | 29.4 | 8.6 | 1.2 | - | - | - | - | - | - |  |
| Soybean | (−6) - (0) | Non-MetS | - | - | - | 68.9 | 3.8 | 2.7 | 24.5 | - | - | - | - | - | - | 0.033 |
|  |  | MetS | - | - | - | 73.6 | 3.0 | 2.0 | 21.4 | - | - | - | - | - | - |  |
| Meat | (−4) - (4) | Non-MetS | - | - | - | - | 1.0 | 2.7 | 6.1 | 11.4 | 78.8 | - | - | - | - | 0.022 |
|  |  | MetS | - | - | - | - | 1.7 | 3.0 | 6.6 | 8.2 | 80.6 | - | - | - | - |  |
| Fish | (−4) - (0) | Non-MetS | - | - | - | - | 17.3 | 13.2 | 69.6 | 0.0 | 0.0 | - | - | - | - | 0.982 |
|  |  | MetS | - | - | - | - | 17.2 | 13.0 | 69.8 | 0.0 | 0.0 | - | - | - | - |  |
| Egg | (−4) - (4) | Non-MetS | - | - | - | - | 32.1 | 31.8 | 15.7 | 13.0 | 7.3 | - | - | - | - | 0.023 |
|  |  | MetS | - | - | - | - | 37.2 | 29.0 | 13.3 | 12.5 | 8.0 | - | - | - | - |  |
| Oil | (0) - (6) | Non-MetS | - | - | - | - | - | - | 68.6 | 31.3 | 0.1 | 0.0 | - | - | - | 0.009 |
|  |  | MetS | - | - | - | - | - | - | 64.9 | 34.7 | 0.4 | 0.0 | - | - | - |  |
| Salt | (0) - (6) | Non-MetS | - | - | - | - | - | - | 0.7 | 35.1 | 58.8 | 5.5 | - | - | - | 0.011 |
|  |  | MetS | - | - | - | - | - | - | 0.5 | 31.5 | 59.9 | 8.1 | - | - | - |  |
| Alcohol | (0) - (6) | Non-MetS | - | - | - | - | - | - | 99.4 | 0.5 | 0.1 | 0.0 | - | - | - | 0.825 |
|  |  | MetS | - | - | - | - | - | - | 99.6 | 0.4 | 0.1 | 0.0 | - | - | - |  |
| Diet variety | (−12) - (0) | Non-MetS | 0.0 | 0.6 | 10.0 | 38.0 | 38.9 | 12.2 | 0.2 | - | - | - | - | - | - | <0.001 |
|  |  | MetS | 0.1 | 1.0 | 12.2 | 42.1 | 37.7 | 6.9 | 0.0 | - | - | - | - | - | - |  |

Abbreviations: MetS, metabolic syndrome.
